# Empirical Examination on the Drivers of the U.S. Equity Returns in the During the COVID-19 Crisis

**DOI:** 10.3389/fpubh.2021.679475

**Published:** 2021-05-21

**Authors:** Qing Wang, Mo Bai, Mai Huang

**Affiliations:** ^1^Department of Finance, Economics and Management School, Wuhan University, Wuhan, China; ^2^School of Accounting, Tianjin University of Commerce, Tianjin, China; ^3^Institute of International Economy, University of International Business and Economics, Beijing, China

**Keywords:** COVID-19 pandemic, stock market returns, US dollar index, volatility index, infectious disease, equity market volatility tracker, least angle regression

## Abstract

This study investigates the drivers of the Standard & Poor's (S&P) 500 equity returns during the COVID-19 crisis era. The paper considers various determinants of the equity returns from December 31, 2019, to February 19, 2021. It is observed that the United States Dollar (USD) and the volatility indices (VIX) negatively affect the S&P 500 equity returns. However, the newspaper-based infectious disease “equity market volatility tracker” is positively associated with the stock market returns. These results are robust to consider both the ordinary least squares (OLS) and the least angle regression (LARS) estimators.

## Introduction

The COVID-19 pandemic has increased the level of uncertainty in every aspect of financial markets, including oil markets (1), commodity markets (2, 3), financial markets (4), energy markets (5), gold markets (6), and stock markets (7–12). Especially after the World Health Organization (WHO) declaration, which states that the COVID-19 pandemic is a global pandemic and will affect millions of people, the United States (U.S.) stock market crashed in March 2020. The S&P 500 equity index has decreased almost 5%, and the Dow-Jones index crashed almost 3,000 points on March 11, 2020. This event was the greatest decline of the U.S. stock markets since Black Monday in 1987 (13, 14).

In an influential paper, Baker et al. (9) show that the COVID-19 crisis causes an unprecedented shock on the U.S. stock markets compared to other pandemics, such as the Spanish flu, Ebola, bird flu, and swine flu. Toda (12) also introduces a susceptible-infected-recovered (SIR) model to estimate the transmission rate of the COVID-19 across the countries. The author also introduces an asset pricing model to predict the stock prices and concludes that the stock market temporarily decreases by 50% in the benchmark scenario. Still, there will be a W-shaped stock market performance in the forthcoming years. Our paper extends this evidence to until mid-February 2021.

Given these backdrops, this research aims to examine the determinants of the U.S. stock returns during the COVID-19 crisis era. We focus on the Standard & Poor's (S&P) 500 daily equity returns from December 31, 2019, to February 19, 2021. Following previous papers, we use the gold returns and crude oil returns to capture the hedging and portfolio diversification purposes in the financial markets (6, 11). We also include the United States Dollar (USD)'s real value to capture the monetary policy's effects on the stock market returns (15–17). We then add the volatility index (VIX) and the newspaper-based infectious disease equity market volatility tracker (EMVT-ID), which contain useful information for modeling stock market returns (18–21).

Although several studies have examined the determinants of the stock market returns in the previous empirical literature, to the best of our knowledge, this is the first research in the literature to examine the determinants of the U.S. stock market returns using a kernel-based estimator. For this purpose, we use the least angle regression (LARS) method of Efron et al. (22) to check the ordinary least squares (OLS) findings' robustness. We find that the USD and the VIX negatively affect the S&P 500 equity returns. However, the newspaper-based EMVT-infectious disease is positively associated with the stock market returns. These results are robust to consider both the OLS and the LARS estimators.

The structure of the remaining parts of the paper is defined as follows. Section Literature Review briefly reviews the previous papers on the determinants of the stock market returns during the COVID-19. Section Empirical Model, Data, and Estimation Methods explains the empirical model, the data, and the estimation procedures. Section Empirical Results reports the empirical results for the OLS estimations with the robust standard errors. Section Robustness Checks discusses various robustness checks, including the LARS estimations' findings with the robust standard errors for different models and periods. Section Concluding Remarks provides the concluding remarks.

## Literature Review

Previous studies examine the determinants of the stock market returns and the stock market price volatility during the COVID-19 era. For instance, Baker et al. (9) consider text-mining approaches to measures the volatility in daily stock market returns to 1900 and the stock market volatility back to 1985. The authors show that there is an unprecedented stock market reaction to the COVID-19 crisis. Alfaro et al. (7) also show that the unexpected changes in the COVID-19 spread indicators can successfully predict the U.S. stock returns using the real-time dataset. Ashraf (8) uses the daily datasets on the COVID-19 confirmed cases and the COVID-19-related deaths to predict the stock market in 64 countries from January 22, 2020, to April 17, 2020. The author observes that the COVID-19 confirmed cases negatively affect the stock market returns. The impact is higher in the COVID-19 confirmed cases than the COVID-19-related deaths. Finally, the results indicate that the negative market reaction to the COVID-19 confirmed cases persists between 40 and 60 days.

Bai et al. (18) use the EMVT-ID to examine the effects of pandemics on stock markets' price volatility in China, Japan, the United Kingdom, and the U.S. for the period from January 2005 to April 2020. The authors observe that a higher level of the EMVT-ID increases the stock market volatility values with a 24-month lag. At this stage, the EMVT-ID provides the smallest impact on the Chinese stock market's price volatility values. Mazur et al. (13) examine the U.S. stock market performance in the market crash of March 2020 due to the COVID-19 shocks. The authors observed significant differences and asymmetries among the stock performances at the sectoral level. Shahzad et al. (23) also confirm this evidence by using the quantile return spillover method. Sharif et al. (11) investigate the causal relationships among the COVID-19 pandemic, economic policy uncertainty, geopolitical risks, oil price, and the U.S. stock market. The wavelet-based approaches' results indicate that the COVID-19 pandemic indicator has a higher impact on the indices of geopolitical risks and economic policy uncertainty than the U.S. stock market returns. Finally, Wang et al. (14) also show that the VIX has the strongest predictive ability for forecasting the futures price volatility in several stock markets during the COVID-19 crisis. The results are robust to consider different volatility methods and to focus on different subsamples. Singh et al. (24) also conclude that there is a temporary negative impact of the COVID-19, and the recovery of stock markets in the G-20 economies started after 60 days from the first shock.

Our previous empirical paper review shows that the COVID-19-related shocks negatively affect stock markets in developed economies. However, the effect is temporary. We also observe that there is no paper in the literature to use the kernel-based estimators. We fill this gap by implementing the LARS estimator.

## Empirical Model, Data, and Estimation Methods

### Empirical Model and Data

We estimate the following model to examine the determinants of the Standard & Poor's (S&P) 500 equity returns:

(1) $$\begin{array}{l} {{lnr\text{\_}S\;\&P500}_{t}=\alpha_{0}+\alpha_{1}X}_{t}+\varepsilon_{t} \end{array}$$

In Equation (1), *lnr* < *uscore* > *S &P*500_*t*_ is the S&P 500 equity index's natural logarithmic (log) returns. The data are obtained from St. Louis FED (25). Following previous literature, we focus on various determinants of the S&P 500 equity index, which are represented by *X*_*t*_. The first indicator is the gold log returns (Fixing Price 3:00 p.m., London Time, in London Bullion Market, based in the USD), and the related data are downloaded by St. Louis FED (26). The second variable is the Brent crude oil log returns, and the related data are obtained from St. Louis FED (27). The third indicator is the trade weighted USD index log returns, and the related data are downloaded by St. Louis FED (27). Note that a higher level of this index means that there is an appreciation in the USD. The fourth indicator is the Chicago Board Options Exchange (CBOE) volatility index (VIX), and the data are obtained from St. Louis FED (27). The VIX measures the market expectation of 3-month volatility conveyed by the S&P 500 stock index option prices. A higher level of the VIX implies that there are higher uncertainty expectations in the U.S. stock markets.

Finally, we use the level change of the newspaper-based Infectious Disease Equity Market Volatility Tracker (EMVT-ID) provided by Baker et al. (28), and the related data are accessed by St. Louis FED (27). The daily frequency data available are from January 1985 up to now. Baker et al. (28) define four terms with their variants to construct the EMVT-ID:

E: {“economic”, “economy”, “financial”},
M: {“stock market”, “equity”, “equities”, “Standard and Poor's”},
V: {“volatility”, “volatile”, “uncertain”, “uncertainty”, “risk”, “risky”},
ID: {“epidemic”, “pandemic”, “virus”, “flu”, “disease”, “coronavirus”, “MERS”, “SARS”, “Ebola”, “H5N1”, “H1N1”}.

Then, Baker et al. (28) provide text mining on daily newspaper articles to check whether there is at least one term in each of *E, M, V*, and *ID* in almost 3,000 United States newspapers. After this, the share of raw EMV-ID articles is calculated in all articles in a given day. Finally, the raw index is rescaled by matching the VIX level between 1990 and 2016 and the overall EMV index (29). Thus, the EMV-ID tracker has been introduced as the ID-EMV articles' ratio to the total EMV articles. Note that a higher level of the index indicates a higher level of pandemic uncertainty in the financial markets (9). Refer to https://www.policyuncertainty.com/infectious_EMV.html for the details of the EMVT-ID index.

Our data focus on the COVID-19 era, i.e., it captures the daily frequency data from December 31, 2019, to February 19, 2021. The period is selected because of data availability. Therefore, we have 276 daily observations. The selection of the period is due to the starting date of the COVID-19 pandemic across the globe. Brief descriptive statistics are provided in Table 1.

**Table 1:** Descriptive statistics (December 31, 2019–February 19, 2021).

**Indicator**	**Definition**	**Data source**	**Mean**	**Std. dev**.	**Min**.	**Max**.	**Obs**.
S&P 500 equity index	Log returns	St. Louis FED (25)	0.00068	0.210	−0.127	0.089	276
Gold price	Log returns	St. Louis FED (26)	0.00055	0.122	−0.054	0.067	276
Oil price	Log returns	St. Louis FED (27)	−0.00027	0.069	−0.643	0.412	276
U.S. dollar index	Log returns	St. Louis FED (30)	−0.00008	0.003	−0.019	0.019	276
Volatility index	Log returns	St. Louis FED (31)	0.00170	0.093	−0.266	0.480	276
Infectious disease EMVT	Change	St. Louis FED (32)	0.07608	8.787	−30.33	30.26	276

Table 1 indicates the positive returns in the S&P 500 equity index and gold market on average. There are negative returns in the crude oil market on average and the USD index, which shows the USD's depreciation. There are positive returns in the uncertainty indices, such as the VIX and the EMVT-ID. In terms of the price volatility, the S&P equity 500 index has a higher standard deviation than the gold and crude oil markets over the period under concern.

Furthermore, the pairwise correlation matrix for the empirical analysis indicators is reported in Table 2 over the period under concern.

**Table 2:** Correlation matrix (December 31, 2019–February 19, 2021).

**Indicator**	**The S&P 500 returns**	**Gold returns**	**Oil returns**	**USD returns**	**VIX returns**	**ΔEMVT-ID**
The S&P 500 returns	1.000	–	–	–	–	–
Gold returns	0.166	1.000	–	–	–	–
Oil returns	0.345	0.0843	1.000	–	–	–
USD returns	−0.399	−0.245	−0.220	1.000	–	–
VIX returns	−0.705	−0.016	−0.266	0.2477	1.000	–
ΔEMVT-ID	0.016	−0.152	−0.030	0.0577	0.0429	1.000

Table 2 shows positive correlations between the S&P 500 equity returns and gold returns, and crude oil returns. The correlations between the S&P 500 equity returns and the USD and the VIX returns are negative. The change in the EMVT-ID is positively related to the S&P equity 500, the USD, and the VIX returns. Simultaneously, gold and crude oil returns are negatively correlated with the change in the EMVT-ID. There are also mixed correlations among the gold, crude oil, and USD returns.

### Estimation Methods

We consider the OLS method to estimate the empirical model in Equation (1). It is important to note that we confirm the stationarity of all indicators by implementing unit root tests. We do not report the related results to save space due to the limited pages. We also implement the LARS method of Efron et al. (22) to check OLS findings' robustness. The LARS is a model-building algorithm that models parsimony and prediction accuracy. This estimation procedure is simpler than the least absolute shrinkage and selection operator (LASSO) and the forward stage-wise regression (FSR). Refer to Efron et al. (22) and Mander (33) for the LARS estimation method's details.

## Empirical Results

The OLS estimations' findings with the robust standard errors for Equation (1) are provided in Table 3.

**Table 3:** Results of the OLS estimations.

**Indicator**	**(1)**	**(2)**	**(3)**	**(4)**	**(5)**
Gold (Log returns)	0.286^***^ (0.102)	0.238^**^ (0.097)	0.110 (0.094)	0.168^**^ (0.070)	0.187^***^ (0.070)
Oil (Log returns)	–	0.101^***^ (0.017)	0.081^***^ (0.016)	0.039^***^ (0.012)	0.039^***^ (0.012)
U.S. dollar index (log returns)	–	–	−1.762^***^ (0.304)	−1.046^***^ (0.231)	−1.050^***^ (0.230)
Volatility Index (log returns)	–	–	–	−0.140^***^ (0.010)	−0.141^***^ (0.010)
Infectious disease EMVT (change)	–	–	–	–	0.017^*^ (0.009)
Adjusted R-squared	0.0242	0.1321	0.2243	0.5705	0.5744
Observations	276	276	276	276	276

*The dependent variable is the S&P 500 equity index log returns. Constant term is included. The robust standard errors are in ()*.

*** *p < 0.01*,

** *p < 0.05, and*

* *p < 0.10*.

Column (1) in Table 3 reports the results for the model with the gold returns. The results indicate that the gold returns are positively related to the S&P 500 equity returns. Column (2) in Table 3 provides the model's findings with the gold and crude oil returns. The findings show that both the gold and crude oil returns are positively related to the S&P 500 equity returns. Column (3) in Table 3 reports the model's results with the gold, crude oil, and USD index returns. The results show that both the gold and crude oil returns are positively associated with the S&P 500 equity returns. However, the USD index returns are negatively related to the S&P 500 equity returns. It is important to note that the gold returns coefficient is statistically insignificant, which means that gold's impact on the S&P 500 equity returns is not robust to include additional controls. Column (4) in Table 3 provides the model's results with the gold, crude oil, USD index, and VIX returns. The findings state that both gold and crude oil returns are positively related to the S&P 500 equity returns. However, the USD index and VIX returns are negatively associated with the S&P 500 equity returns. Note that all coefficients are statistically significant.

Finally, Column (5) in Table 3 reports the findings for the model with the gold, crude oil, USD index, and VIX returns and the change in EMVT-ID. The results show that the gold returns, the crude oil returns, and the changes in the EMVT-ID are positively related to the S&P 500 equity returns. However, the USD index and VIX returns negatively affect the S&P 500 equity returns. Again, all coefficients are statistically significant.

We also find the adjusted R-squared as 0.5744, which means that the gold, crude oil, USD index, and VIX returns and the changes in the infectious disease EMVT explain 57.44% of the S&P 500 equity returns. Overall, the OLS estimations' findings indicate that the crude oil returns and the changes in the infectious disease EMVT positively affect the S&P 500 equity returns. In contrast, the USD index and VIX returns are negatively related to the S&P 500 equity returns. The average coefficients of the crude oil, USD index, and VIX returns and the infectious disease EMVT are calculated as 0.065, −1.286, −0.140, and 0.017, respectively.

## Robustness Checks

### The LARS Estimations

The LARS estimations with the robust standard errors for Equation (1) are reported in Table 4.

**Table 4:** Results of the LARS estimations.

**Indicator**	**(1)**	**(2)**	**(3)**	**(4)**	**(5)**
Gold (log returns)	0.028 (0.035)	0.005 (0.081)	0.049 (0.081)	0.003 (0.071)	0.024 (0.063)
Oil (log returns)	–	0.079^***^ (0.020)	0.061^***^ (0.019)	0.031 (0.023)	0.025 (0.020)
U.S. dollar index (log returns)	–	–	−0.643^**^ (0.265)	−0.496^**^ (0.238)	−0.456^**^ (0.215)
Volatility Index (log returns)	–	–	–	−0.104^***^ (0.011)	−0.102^***^ (0.009)
Infectious disease EMVT (change)	–	–	–	–	0.020^**^ (0.008)
Adjusted R-squared	0.0059	0.0878	0.1336	0.6455	0.7197
Observations	276	276	276	276	276

*The dependent variable is the S&P 500 equity index log returns. Constant term is included. The robust standard errors are in ()*.

*** *p < 0.01*,

** *p < 0.05*.

Column (1) of Table 4 provides the findings for the basic model with the gold returns. The results indicate that the gold returns are positively associated with the S&P 500 equity returns. However, its coefficient is not statistically significant. Column (2) in Table 4 indicates the model's results with the gold and crude oil returns. The results show that both the gold and crude oil returns are positively associated with the S&P 500 equity returns. However, the coefficient of the gold returns is statistically insignificant. Column (3) in Table 4 presents the model's findings with the gold, crude oil, and USD index returns. The results show that both the gold and crude oil returns are positively related to the S&P 500 equity returns. Besides, the USD index returns negatively affect the S&P 500 equity returns. At this point, the coefficient of the gold returns is not statistically significant. Column (4) in Table 4 provides the model's findings with the gold, crude oil, USD index, and VIX returns. The findings illustrate that both the gold and crude oil returns are positively related to the S&P 500 equity returns. However, their coefficients are not statistically significant, which means that the effects of the gold and crude oil returns on the stock market returns are not robust to consider different models and different estimators. At this stage, the USD index and VIX returns negatively affect the S&P 500 equity returns, and their effects are statistically significant at the 5% level at least.

Finally, Column (5) in Table 4 provides the results for the model with the gold, crude oil, USD index, and VIX returns and the change in infectious disease EMVT. The findings are that both the gold and crude oil returns and the changes in the infectious disease EMVT are positively related to the S&P 500 equity returns. However, merely the coefficient of the infectious disease EMVT is statistically significant at the 5% level. Furthermore, the USD index and VIX returns are negatively associated with the S&P 500 equity returns. Similarly, the related coefficients are statistically significant at the 5% level at least.

We also obtain the adjusted R-squared as 0.7197, implying that the gold, crude oil, USD index returns, and VIX returns and the changes in the infectious disease EMVT explain 71.97% of the S&P 500 equity returns. The adjusted R-squared of the LARS estimations is significantly higher than that of the OLS estimations. In short, the results of the LARS estimations show that the changes in the infectious disease EMVT positively affect the S&P 500 equity returns. However, the USD index and VIX returns are negatively associated with the S&P 500 equity returns. The average coefficients of the USD index returns, the VIX returns, and the change in the infectious disease EMVT are found as −0.531, −0.103, and 0.020, respectively.

### The LARS Estimations With Lagged Model

We have also considered the lagged indicators for the gold, crude oil, USD index, and VIX returns and the change in infectious disease EMVT. The related results are provided in Table 5.

**Table 5:** Results of the LARS estimations (lagged model).

**Indicator**	**(1)**	**(2)**	**(3)**	**(4)**	**(5)**
Lagged gold (log returns)	0.119 (0.102)	0.074 (0.097)	0.046 (0.094)	0.071 (0.069)	0.028 (0.069)
Lagged oil (log returns)	–	0.093^***^ (0.016)	0.074^***^ (0.015)	0.031^***^ (0.011)	0.034^***^ (0.011)
Lagged U.S. dollar index (log returns)	–	–	−1.663^***^ (0.287)	−0.983^***^ (0.217)	−0.989^***^ (0.215)
Lagged volatility index (log returns)	–	–	–	−0.133^***^ (0.009)	−0.134^***^ (0.008)
Lagged infectious disease EMVT (Change)	–	–	–	–	0.023^***^ (0.008)
Adjusted R-squared	0.0049	0.1133	0.2018	0.5623	0.5714
Observations	275	275	275	275	275

*The dependent variable is the S&P 500 equity index log returns. Constant term is included. The robust standard errors are in ()*.

*** *p < 0.01*.

Column (1) of Table 5 shows the results for the simple model with the lagged gold returns. The results show that the lagged gold returns are positively related to the S&P 500 equity returns. However, its coefficient is not statistically significant. Column (2) in Table 5 states the model's findings with the lagged gold and lagged crude oil returns. The results indicate that both the lagged hold and lagged crude oil returns are positively associated with the S&P 500 equity returns. However, the coefficient of the lagged gold returns is statistically insignificant. Column (3) in Table 5 demonstrates the model's results with the lagged gold, lagged crude oil, and lagged USD index returns. The findings indicate that both the lagged gold and lagged crude oil returns are positively associated with the S&P 500 equity returns. Besides, the lagged USD index returns negatively affect the S&P 500 equity returns. At this point, the coefficient of the gold returns is not statistically significant. Column (4) in Table 5 reports the model's findings with the lagged gold, lagged crude oil, lagged USD index, and lagged VIX returns. The results show that both the lagged gold and lagged crude oil returns are positively associated with the S&P 500 equity returns. However, the coefficient of the lagged gold returns is not statistically significant. At this point, the lagged USD index and lagged VIX returns negatively affect the S&P 500 equity returns, and their effects are statistically significant at the 1% level.

Finally, Column (5) in Table 5 demonstrates the findings for the model with the lagged gold, lagged crude oil, lagged USD index, and lagged VIX returns and the lagged change in the infectious disease EMVT. The findings are that the lagged gold and lagged crude oil returns and the lagged changes in the infectious disease EMVT are positively associated with the S&P 500 equity returns. The coefficients of the lagged infectious disease EMVT and the lagged crude oil returns are statistically significant at the 1% level. At this stage, the lagged USD index and lagged VIX returns are negatively associated with the S&P 500 equity returns. Similarly, the related coefficients are statistically significant at the 1% level.

We also observe the adjusted R-squared as 0.5714, implying that the lagged gold, lagged crude oil, lagged USD index, and lagged VIX returns and the lagged changes in the infectious disease EMVT explain 57.14% of the S&P 500 equity returns. Overall, the LARS estimations' findings with the lagged model indicate that the lagged changes in the infectious disease EMVT and the lagged crude oil positively affect the S&P 500 equity returns. However, the lagged USD index and lagged VIX returns are negatively related to the S&P 500 equity returns. The average coefficients of the lagged USD index returns, the lagged VIX returns, and the lagged change in the infectious disease EMVT are obtained as −1.211, −0.133, and 0.023, respectively. These results are in line with the benchmark LARS estimations in Table 4 and Column 5.

### The LARS Estimations for the Subperiods

We also provide additional robustness checks. We have analyzed the results in various subperiods. At this stage, we follow the break dates in Dong et al. (34) and Zhang et al. (35). On March 13, 2020, there is the declaration of a national emergency. On March 27, 2020, the Coronavirus Aid, Relief, and Economic Security (CARES) was enacted. The stimulus package started on April 15, 2020. Therefore, we focus on the period from April 15, 2020, to February 19, 2021. These findings are reported in Table 6.

**Table 6:** Results of the LARS estimations: April 15, 2020–February 19, 2021 (lagged model).

**Indicator**	**(1)**	**(2)**	**(3)**	**(4)**	**(5)**
Lagged gold (log returns)	0.080 (0.086)	0.084 (0.086)	0.021 (0.018)	0.048 (0.075)	0.025 (0.015)
Lagged oil (log returns)	–	0.023^***^ (0.008)	0.033^***^ (0.009)	0.032^***^ (0.010)	0.038^***^ (0.010)
Lagged U.S. dollar index (log returns)	–	–	−0.557^***^ (0.189)	−0.492^***^ (0.183)	−0.517^***^ (0.201)
Lagged volatility index (log returns)	–	–	–	−0.134^***^ (0.015)	−0.131^***^ (0.014)
Lagged infectious disease EMVT (Change)	–	–	–	–	0.024^***^ (0.009)
Adjusted R-squared	0.0042	0.1224	0.1554	0.3173	0.3362
Observations	205	205	205	205	205

*The dependent variable is the S&P 500 equity index log returns. Constant term is included. The robust standard errors are in ()*.

*** *p < 0.01*.

Column (1) of Table 6 indicates the findings for the basic model with the lagged gold returns. The findings demonstrate that the lagged gold returns are positively related to the S&P 500 equity returns. However, its coefficient is insignificant. Column (2) in Table 6 reports the findings with the lagged gold and lagged crude oil returns. The findings indicate that both the lagged gold and lagged crude oil returns are positively related to the S&P 500 equity returns. However, the coefficient of the lagged gold returns is not statistically significant. Column (3) in Table 6 indicates the model's results with the lagged gold, lagged crude oil, and lagged USD index returns. The results show that both the lagged gold and lagged crude oil returns are positively related to the S&P 500 equity returns. Besides, the lagged USD index returns negatively affect the S&P 500 equity returns. At this stage, the coefficient of the gold returns is statistically insignificant. Column (4) in Table 6 provides the model's results with the lagged gold, lagged crude oil, lagged USD index, and lagged VIX returns. The findings indicate that both the lagged gold and lagged crude oil returns are positively related to the S&P 500 equity returns. However, the coefficient of the lagged gold returns is statistically insignificant. Here, the lagged USD index and lagged VIX returns negatively affect the S&P 500 equity returns, and their effects are statistically significant at the 1% level.

Finally, Column (5) in Table 6 reports the results for the model with the lagged gold, lagged crude oil, lagged USD index, and lagged VIX returns and the lagged change in the infectious disease EMVT. The results are that both the lagged gold and lagged crude oil returns and the lagged changes in the infectious disease EMVT are positively related to the S&P 500 equity returns. The coefficients of the lagged infectious disease EMVT and the lagged crude oil returns are statistically significant at the 1% level. Here, the lagged USD index and lagged VIX returns negatively affect the S&P 500 equity returns. Similarly, the related coefficients are statistically significant at the 1% level.

We also observe the adjusted R-squared as 0.3362, implying that the lagged gold, lagged crude oil, lagged USD index, and lagged VIX returns and the lagged changes in the infectious disease EMVT explain 33.62% of the S&P 500 equity returns. Overall, the LARS estimations' results with the lagged model on subperiod show that the lagged changes in the infectious disease EMVT and the lagged crude oil positively affect the S&P 500 equity returns. However, the lagged USD index and lagged VIX returns are negatively associated with the S&P 500 equity returns. The average coefficients of the lagged USD index returns, the lagged VIX returns, and the lagged change in the infectious disease EMVT are obtained as −0.522, −0.132, and 0.024, respectively. These findings are in line with the benchmark LARS estimations in Table 4 and Column 5.

## Concluding Remarks

This study examines the S&P 500 equity index returns determinants from December 31, 2019, to February 19, 2021. The empirical results show that the USD and the VIX are negatively associated with the S&P 500 equity index returns. However, the newspaper-based infectious disease “equity market volatility tracker” positively affects the S&P 500 equity index returns. These results are robust to estimate both the OLS and the LARS method of Efron et al. (22). Future papers in this subject can focus on other financial assets, including cryptocurrencies, for investigating the determinants of financial assets' returns and the price volatility.

Given that our findings are limited with the daily-frequency data, particularly, intraday data can provide additional findings for the effects of the COVID-19-related uncertainties on cryptocurrencies and financial markets. Following Sharif et al. (11), we suggest that the index of geopolitical risks be included in a future study. Geopolitical risks can divert people's attention from the government's ineffective response to the COVID-19 pandemic, and therefore, they can negatively affect the stock market performance.

## Data Availability Statement

Publicly available datasets were analyzed in this study. This data can be found here: https://fred.stlouisfed.org/series.

## Author Contributions

QW: writing original draft and estimations. MB: writing original draft. MH: data collection and reviewing the original draft. All authors contributed to the article and approved the submitted version.

## Conflict of Interest

The authors declare that the research was conducted in the absence of any commercial or financial relationships that could be construed as a potential conflict of interest.
